# Random Texts Do Not Exhibit the Real Zipf's Law-Like Rank Distribution

**DOI:** 10.1371/journal.pone.0009411

**Published:** 2010-03-09

**Authors:** Ramon Ferrer-i-Cancho, Brita Elvevåg

**Affiliations:** 1 Departament de Llenguatges i Sistemes Informàtics, Universitat Politècnica de Catalunya, Barcelona, Catalonia, Spain; 2 Clinical Brain Disorders Branch, National Institute of Mental Health, National Institutes of Health, Bethesda, Maryland, United States of America; University of East Piedmont, Italy

## Abstract

**Background:**

Zipf's law states that the relationship between the frequency of a word in a text and its rank (the most frequent word has rank 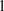, the 2nd most frequent word has rank 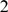,…) is approximately linear when plotted on a double logarithmic scale. It has been argued that the law is not a relevant or useful property of language because simple random texts - constructed by concatenating random characters including blanks behaving as word delimiters - exhibit a Zipf's law-like word rank distribution.

**Methodology/Principal Findings:**

In this article, we examine the flaws of such putative good fits of random texts. We demonstrate - by means of three different statistical tests - that ranks derived from random texts and ranks derived from real texts are statistically inconsistent with the parameters employed to argue for such a good fit, even when the parameters are inferred from the target real text. Our findings are valid for both the simplest random texts composed of equally likely characters as well as more elaborate and realistic versions where character probabilities are borrowed from a real text.

**Conclusions/Significance:**

The good fit of random texts to real Zipf's law-like rank distributions has not yet been established. Therefore, we suggest that Zipf's law might in fact be a fundamental law in natural languages.

## Introduction

Imagine that one takes a text, counts the frequency of every word and assigns a rank to each word in a decreasing order of frequency. This would result in the most frequent word having a rank of 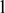, the second most frequent word having a rank of 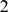 and so on. The histogram of such word ranks is said to conform to Zipf's law for word frequencies [1]. In its simplest form, the law states that 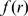, the frequency of a word or rank 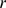 obeys
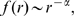
(1)where 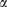 is a constant, the so-called exponent of the law (typically 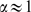 [1]). In other words, Eq. 1 indicates that frequency decays linearly as the rank increases on double logarithmic scale. Although the law was originally thought to reveal principles of language functioning [1], many have argued against its relevance [2]–[7]. Their major claim is that the statistics of simple random sequences of characters - including a special one that behaves as a word delimiter - reproduces Zipf's law for word frequencies [2], [4], [5]. Henceforth, we refer to this special character as a space or a blank. For instance, the random text

*wbqcrw h q rorjleabeyxkrlpqkpchnesguliwkb mrltn q a rss vfs w a h rlzpxxtxbkqetfwfpqudgwaorqwgqmo wyngwtbseuodboxaw x rldua eucx mmard xgqzv uu pueuerc pkizuauyrwi bllhjddv bp anud xbxvjyymioymvzebc tdtsecdijntssyepqdubcvxjd evavybwvejp w z uvspufvdvuzyf t nllifznwatic*

has been generated using English letters ranging from ‘a’ to ‘z’ (the separation between words in our example is arbitrary and due to automatic formatting).

The idea that random sequences of characters reproduce Zipf's law stems from the seminal work of Mandelbrot [8] and was reformulated in various works [2], [4], [5], [9]. We refer to a random sequence of characters of the type listed above as a random text so as to be consistent with [2] although more appropriate names have been discussed [10]. The simplest version of a random text is based upon the assumption that all characters are equally likely [2], [7]. We define 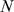 as the number of regular characters of the random text and 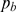 as the probability of a blank. The above example was generated with 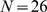 and 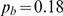, which was deemed suitable for English [4], [5]. It is noteworthy that when constructing the example above, we assumed that all characters are independent, that all letters from ‘a’ to ‘z’ are equally likely and two or more blanks in a row are not permitted. If two blanks in a row are not allowed then words with no characters (i.e. empty words) cannot be generated.

There have been many arguments against the meaningfulness or relevance of Zipf's law [2], [4], [6], [7]. However, there are also reasons that such arguments might be flawed:

- *Problem 1*
  The studies that question the relevance to natural language of Zipf's law argue for the matching between Eq. 1 and random texts. However, Eq. 1 is only an approximation for the real rank histogram. The best candidate for the actual rank distribution remains an open question [11]–[14] for two reasons: first, the goodness of the fit provided by Eq. 1 in a statistically rigorous sense is questionable and, second, the best function may not be unique [15]. If it turned out that when using statistically rigorous methods that real texts do not usually fit Eq. 1, then the arguments against the relevance of Zipf's law would be seriously challenged.
- *Problem 2*
  As far as we know, in none of the popular articles that argue against the meaningfulness of Zipf's law [2]–[7] is there an accurate enough derivation of Zipf's law (Eq. 1) from random texts. This is of crucial importance because real texts and random texts may seem to have consistent rank distributions if not regarded with enough precision simply because two distinct tiny objects may look similar if our lens is not powerful enough. Notice that in [2]–[7] an exact derivation of Zipf's law from the assumptions of a random text is absent. Instead, only equations that are valid for the ensemble of words of a certain length are provided. For instance, Li [2] defines 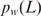 as the probability of any particular word of length 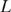 and proves that
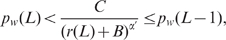
(2)where 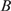, 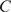 and 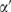 are constants and 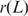 is the rank of any word of length 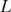 (a similar derivation can be found in [7]). Miller & Chomsky [4] showed that the probability of any word of length 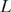 obeys
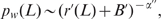
(3)where 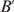 and 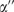 are constants and 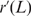 is now the mean rank of all the possible words of length 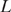. In contrast, notice that Zipf's law (Eq. 1) is a law of individual ranks, not a law of a rank chosen to represent all words of the same length (e.g., the average rank or words of the same length). Recently, it has been proven that 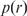, the probability of observing a word of rank 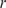 in a random text, obeys [16], for sufficiently large 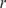,
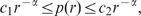
(4)where 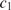 and 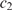 are two positive constants. Although the derivation of Eq. 4 in [16] for a general class of random texts is a milestone in the history of random texts, notice that Eq. 4 is weaker than the definition of Zipf's law in Eq. 1.
- *Problem 3*
  Eqs. 2, 3 and 4 are derived in the context of a very long text. It is not known *a priori* if the parameters of the underlying exact distribution of ranks depend upon the text length or if the distribution that is obtained in the context of a very long text is the same as that of a random text of the size of the order of real texts.
- *Problem 4*
  As far as we know, in none of the popular articles that question the meaningfulness to natural language of Zipf's law [2]–[7] is there any comparison between the rank histograms of actual texts and those of random texts. Rather it is simply taken for granted that an approximate agreement with Eq. 1 is sufficient. To the best of our knowledge, in none of these cases is either a visual comparison between the rank histogram of a real text and that of a random text provided (e.g., by plotting both histograms together), nor are more convenient rigorous tests of the goodness of fit of random texts for real texts performed. In some exceptional cases, a visual comparison between a real text and an equation similar to Eq. 3 has been made [17] but the comparison implies the misuse of an equation that was originally derived for the mean rank of words of the same length to the individual ranks of actual Zipf's law-like rank distributions. Although Mandelbrot did not show simultaneously real and artificial rank distributions, arguably he inappropriately used equations that had been derived for individual ranks (e.g., Fig. 1 of [18] and Fig. 2 of [8]).

**Figure 1:**
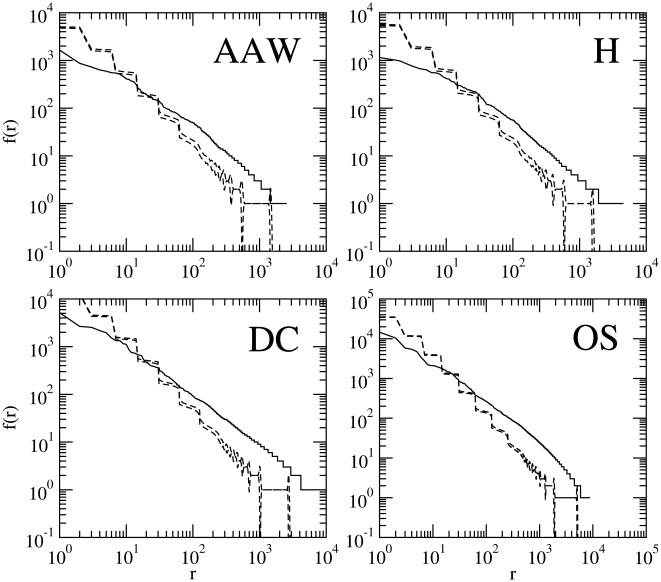
The rank histograms of English texts versus that of random texts (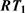). A comparison of the real rank histogram (thin black line) and two control curves with the 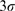 upper and lower bounds of the expected histogram of a random text of the same length in words (dashed lines) involving four English texts. 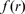 is the frequency of the word of rank 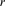. For the random text we use the model 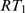 with alphabet size 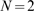. The expected histogram of the random text is estimated averaging over the rank histograms of 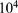 random texts. For ease of presentation, the expected histogram is cut off at expected frequencies below 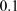. AAW: *Alice's adventures in wonderland*. H: *Hamlet*. DC: *David Crockett*. OS: *The origin of species*.

**Figure 2:**
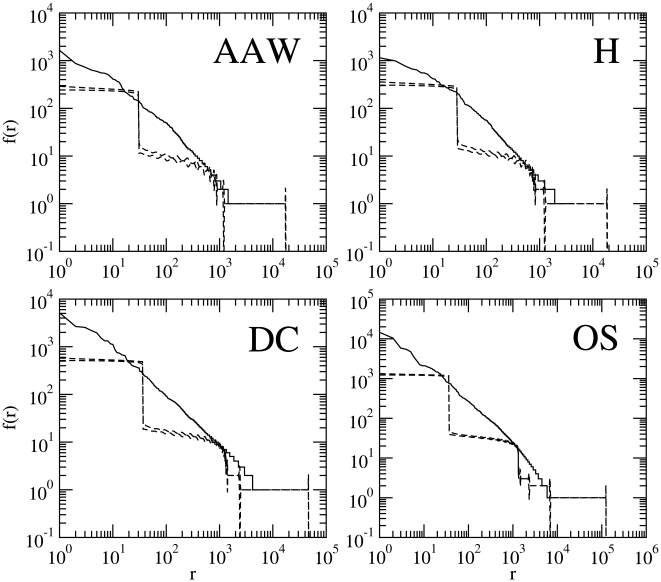
The rank histograms of English texts versus that of random texts (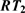). The same as Fig. 1 for the model 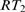 with alphabet size 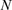 and probability of blank 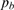 obtained from the real text.

To address Problem 1, we evaluate the goodness of fits of random texts to real texts *directly* by means of samples of ranks produced by the real process and not *indirectly* through Eq. 1. To address Problem 2 we study the consistency between rank samples from a random text and rank samples from a real text using three rigorous statistical tests. We skip the mathematical challenge of obtaining the missing exact rank distribution for individual ranks. To address Problem 3, we compare real texts with random texts of the same length. In this way, we can establish that putative differences cannot be attributed to simply differences in the text length. To address Problem 4, we compare visually the rank histogram of random texts with those of real texts so as to provide an estimate of the enormous differences between both and then we perform rigorous statistical tests to show that the real word rank histograms are inconsistent with those of random texts.

We exclude from our analysis a variant of the random text that generates empty words. Empty words are obtained when producing two blanks in a row, which is allowed in [4]–[7], [16] but not permitted in Li's version [2] (see Text S1). In other cases, it is not clear if empty words are allowed, e.g., [19]. Excluding empty words in our study is justified by the fact that the goal of this article is to evaluate the fit of random texts for real Zipf's law-like word rank distributions. As far as we know, in none of Zipf's pioneering works [1], [20] and in the many studies that followed, have empty words been included or even considered in real rank histograms. Indeed, their existence in real texts is very questionable.

Many authors have discussed the explanatory adequacy of random texts for real Zipf's law-like word rank distributions indirectly from inconsistencies between random texts and real texts beyond the distribution of ranks [19], [21]–[24]. One of the most typical and recurrent examples is the claim that real word lengths are not geometrically distributed as expected from a random text experiment [21]–[24]. However, the question that we seek to address here is: do random texts really fit the real Zipf's law-like distribution accurately as suggested by many [2], [4]–[7], [25]?

To our knowledge, only a few studies have addressed this question [19], [26], [27] but in a qualitative manner and only for certain versions of the random text model. In this article, we go a step forward by bringing rigorous statistical tests into the debate and considering all the variants of the random text model that have been considered in the literature. In particular, we compare visually some rank histograms from English texts with those of different versions of the random text model and test rigorously the goodness of fit of random texts on actual histograms in a set of ten texts. We demonstrate that - contrary to what has previously been suggested - random texts fail to fit actual texts even visually. Finally, we shed light on the failure of random texts to fit actual texts from the perspective of cognitive science and discuss the implications of our negative results for the meaningfulness to natural language of Zipf's law.

## Results

In this article we employ a set of ten English texts (eight novels and two essays) to evaluate the goodness of fit of random texts in Table 1. A summary of their statistical properties is shown in Table 2.

**Table 1:** Summary of English texts employed.

Title	Abbreviation	Author
Alice's adventures in wonderland	AAW	Lewis Carroll (1832–1898)
The adventures of Tom Sawyer	ATS	Mark Twain (1835–1910)
A Christmas carol	CC	Charles Dickens (1812–1870)
David Crockett	DC	John S. C. Abbott (1805–1877)
An enquiry concerning human understanding	ECHU	David Hume (1711–1776)
Hamlet	H	William Shakespeare (1564–1616)
The hound of the Baskervilles	HB	Sir Arthur Conan Doyle (1859–1930)
Moby-Dick: or, the whale	MB	Herman Melville (1819–1891)
The origin of species by means of natural selection	OS	Charles Darwin (1809–1882)
Ulysses	U	James Joyce (1882–1941)

The data set of English texts employed in our study.

**Table 2:** Statistics of the English texts.

Abbreviation	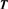 (in words)	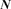 (in chars.)	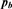	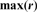	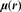	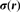
AAW	27342	28	0.254	2574	254.05	466.60
CC	29253	30	0.240	4263	463.31	887.22
H	32839	28	0.253	4582	474.39	932.44
ECHU	57958	36	0.212	4912	433.91	861.35
HB	59967	39	0.244	5568	472.87	990.44
ATS	73523	31	0.248	7169	612.45	1298.53
DC	78819	36	0.228	7385	668.60	1346.19
OS	209176	36	0.207	8955	589.94	1274.53
MB	218522	36	0.229	17190	1291.67	2909.44
U	269589	36	0.228	29213	2425.63	5444.95

Statistical properties of the English texts. See Table 1 for the meaning of each abbreviation. Texts are sorted by increasing length. 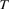 is the text length in words. 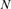 is the number of different characters excluding the blank. 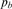 is the estimated probability of blank. 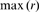 is the maximum rank or the observed vocabulary size. 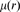 and 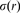 are, respectively, the mean and the standard deviation of the rank.

### The Versions of the Random Text

We consider three different versions of the random text (RT) model without empty words that have been considered in the literature. All the versions generate a random sequence of independent characters. These three version are (the subindex indicates the number of parameters of the version of the random text):

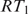

  All characters, including the blank are equally likely. This model is specified with a single parameter: 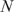, the number of characters other than space. 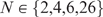 was used in [2]. 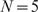 was used by [7] allowing empty words. An example of 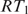 with 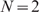 is
*uu kuuuuk k kkk uu u kkuuukuuk uk kukukuuu u ukku kukkk uku uku ku u kuk kukk uuuk k kk kku uuu u kuukkuk u kku kuukuu u uukk ku uuk kukk u ukkkkuuu k ukku kuku kuk k k uku k uuku uu kuukukuukk kukku k uk u*

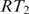

  All characters except the blank are equally likely. This model is specified with two parameters, 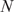 as in 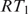 plus 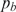, the probability of blank for the 2nd and following characters of a word (notice that in our case, the probability that the current word under construction has no character when the blank is produced is zero). Allowing empty words, 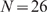 and probability of blank 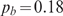 was argued to be suitable for English [4], [5] without explaining how 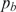 was estimated. Here we obtain 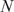 and 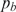 from real normalized texts (see Materials and Methods for details about our text normalization). 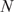 is obtained from the number of different characters of the text (except the blank). 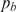 is computed from the formula
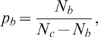
(5)where 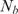 is the number of blanks and 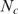 is the total number or characters (including blanks). In our text normalization, 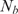 is equivalent to the number of different words (i.e. the maximum rank). 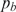 is the proportion of blanks after excluding the first character of each word, which cannot be a blank in our versions of the random text model. An example of 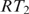 with 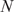 and 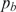 borrowed from *Alice's adventures in wonderland* is
*i 0xbple f h gxadchrdcty hz trsykj o b axurvg qfu k kg3kx vwzsj3 xw0t3f nq ryb uhibb nqhtqb zfgnfk v gdq p30ajh 30 c p k3cgozfe3vt hdmzc k0q bw fs c kgu lm0tx bh av eu v cmbosjbis a3aks mucjtefrtvko t uyprnz eyti h3do hm0mx w0kbecyd ti v qoyowzcfiykv3wb*

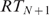

  All characters can take any probability. This model is specified with 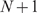 parameters (i.e. the 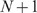 probabilities of each of the characters). Three parameter settings have been considered in the literature:

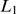

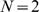
 with the probability of the two characters other than space being 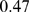 and 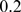 and the probability of space is 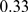 [2].

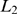

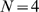
 with the probability of the four characters other than space being 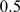, 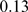, 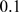 and 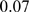 and the probability of space being 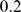 [2].

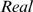

    Real character probabilities extracted from the target writing as in [19].
  An example of 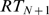 with real character probabilities borrowed from *Alice's adventures in wonderland* is
*tel g shs oo fagl t ersu fa r esnrlaod k ni ihe a o e sh foie r do aorhdaev aiot t oseldtiyie wq t thsynt w e sptsnsm heooeat utdgeco a iyeb sniemt ehdoy t thruw twaame eatendeisidle mc nlhtt ih a utfd anulbgleta nlh ohe gt eehitofnet*

### Visual Fitting

Here we aim to compare rank histograms from real texts and expected rank histograms from random texts. If random texts really reproduce the rank histogram of real texts, then the histogram of real texts and those of the random texts should completely overlap. We will see that this is not the case.

Here our emphasis is on providing a fair visual comparison. We use the term fair in two senses. First, we consider real and artificial texts of the same length in words. Notice that the equations that have been derived so far for the rank distribution of 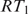 and 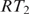 texts are derived in the limit of a very large text in which all words of the same length must have the same frequency of occurrence because they are *a priori* equally likely [2], [4], [5], [7]. If the text is not long enough, the frequency of words of the same length may differ noticeably. Here we aim to equate the text size of both the model and the real text. Second, we do not misuse a theoretical equation that is not valid for individual ranks as in [17]. The theoretical rank distribution or even the theoretical expected rank histogram of random texts are not available, even in their most simples versions. Therefore, we work on the expected rank histogram of random texts, which can be easily estimated by simulating the process and averaging the rank histogram over a sufficiently large number of artificial texts. Third, we do not use binning as in [26] which could shadow the differences between actual texts and random texts.

In the interest of being concise, for visual fits, we chose four works representing different genres and covering the whole range of text lengths in the sample. Fig. 1 shows the rank histogram of the four selected English texts versus the expected rank histogram of a 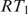. From visual inspection, it is obvious that the agreement between the random text and the real text is poor. The histograms of random texts are clearly above the corresponding real histograms for small ranks and clearly below for larger ranks. Additionally, the curves of real histograms are smoother as compared to the pronounced staircase decrease of random texts, especially for small ranks. Fig. 2 shows that 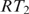 with 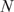 and 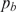 taken from the real text does not improve the quality of the visual fit. The staircase decrease of the histogram of random texts becomes more radical and the plateaus are huge as compared to those of Fig. 1. One may infer from Figs. 1 and 2 that 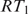 gives a better fit than 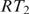 in general, but the difference in fitting is mainly due to the small value of 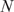 employed in Fig. 1, which produces smaller plateaus with regard to Fig. 2.

**Figure 3:**
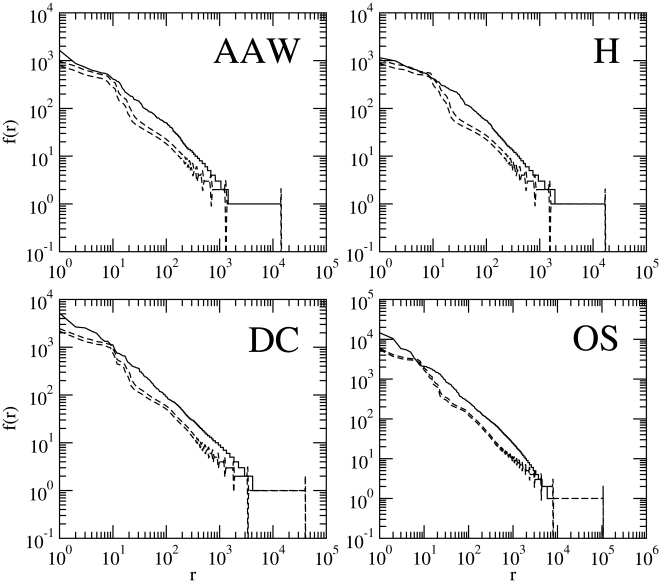
The rank histograms of English texts versus that of random texts (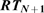). The same as Fig. 1 for the model 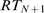 with alphabet size 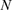 and character probabilities obtained from the real text.

It is well known that if characters other than the blank have unequal probabilities then the rank histogram smoothes [2], [25], [28]. The point is: would this apparently dramatic improvement be enough to achieve a perfect fit? Fig. 3 shows that these random texts (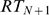 model) still deviate from the real texts from which they borrow the character probabilities. For instance, Fig. 3 shows that random texts display pronounced humps for high frequencies and wider plateaus in the low frequency domain with regard to the real text. Besides, the rank histogram of random texts is clearly longer than that of real texts in Figs. 2 and 3. The fact that the plateaus at the low frequency region are much broader for 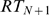 texts than for real texts is well-known [19]. In the next section we show that the differences between real texts and random texts with non-commensurate character probabilities are statistically significant as well as for all the parameters suggested in the literature for 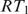 and 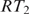.

In the next section, we employ rigorous statistical fitting, not because we think that it is strictly necessary when large visual differences between random and real texts are found (e.g., Figs. 1 and 2), but so as to provide a foundation for a more mathematically precise understanding of the differences between real texts and random texts and to extend, in a concise way, the analysis to the texts and parameters settings not considered in the figures. Notice that the poor visual fit of random texts shown in Figs. 1, 2 and 3 also applies to the real texts in Table 1 not visually examined in these figures so as to conserve space.

### Rigorous Statistical Fitting

We detailed in the introduction that we did not seek to evaluate the goodness of fits of random texts for actual rank histograms through Zipf's law because this implies the risk that the target equation, i.e. Eq. 1, is not accurate enough [11], [14]. A typical way of testing the fit of a certain model to real data is from the exact distribution that characterizes the model [12]. However, as mentioned in the introduction, this is impossible in the current situation because the exact rank distribution of random texts is unknown. To our knowledge, only approximations have been derived. Furthermore, the intention of our article was not to derive this equation *per se*. In light of the absence of such an exact distribution, we evaluate the consistency of ranks from a real text with those of a random text of the same length (in words) through three different statistics of the rank 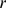 in a text of length 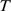:

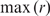
, the maximum rank. 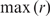 is the observed vocabulary size and measures the width of the rank histogram). Notice that the actual vocabulary of a random text model is infinite (*a priori*, any string of at least one letter can be formed), contrary to the actual vocabulary of a writer, which although large is finite. Support for 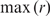 comes from previous work suggesting that the pattern of observed vocabulary growth is useful for distinguishing between natural and 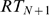 texts [19]. We find that 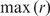 is indeed useful for any kind of random text and that simply its value is enough to distinguish random texts from real texts for the versions and parameter settings considered in this article.

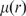
, the mean rank.

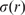
, the standard deviation of the rank.

To our knowledge, the expectation of these statistics for a text of a certain finite length has not previously been reported. If the rank distribution of the real texts and that of the random texts are the same, statistically significant differences between the value of the above statistics in real texts and those of random texts should not be found or be exceptional. Here we consider the whole set of ten English texts including the four works we examined in detail in the previous section (Table 1).

For each real text, we estimate the expectation and standard deviation of these statistics by generating 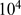 independent random texts for all the versions and parameters of the random text reviewed above. Notice that the length in words of the random texts is the same as that of the real text. Then we calculate 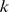, the distance to the mean (measured in units of the standard deviation) between the value of real value of the statistic in the target text and that of a random text of a certain version and parameter setting. The three rank statistics yield three distances, i.e.,
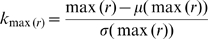
(6)

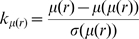
(7)

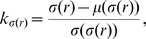
(8)The sign of the distance indicates whether the actual value is smaller than the expected (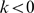) or larger than expected (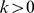) for the hypothesis of a random text. Table 3 shows a summary of these signed distances for the texts in our data set.

**Table 3:** Distance to the mean in standard deviations.

				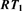			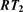		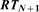	
Abbrv.	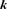	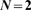	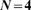	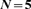	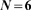	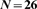	-	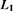	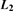	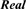
AAW	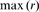	42.6	−97.5	−133.2	−163.4	−573.4	−160.6	54.0	−74.3	−147.1
	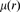	130.5	−59.7	−78.6	−94.2	−312.9	−93.1	173.0	−46.3	−85.7
	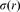	56.3	−83.4	−119.5	−156.8	−2033.6	−153.1	74.1	−63.0	−135.5
CC	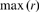	99.1	−80.7	−120.0	−151.1	−555.4	−158.5	116.8	−53.7	−139.3
	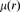	267.3	−54.5	−76.8	−93.6	−317.8	−98.0	347.2	−36.8	−87.1
	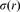	136.5	−72.8	−111.9	−149.5	−1969.6	−159.2	169.9	−48.7	−134.7
H	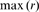	103.5	−86.6	−127.6	−158.4	−581.5	−157.7	121.5	−58.6	−142.3
	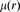	277.8	−58.4	−81.4	−97.6	−331.3	−97.5	361.9	−40.5	−89.1
	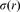	142.1	−77.8	−118.3	−155.8	−2017.9	−154.6	176.8	−53.1	−135.4
ECHU	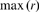	75.6	−133.8	−184.6	−226.0	−795.6	−275.9	93.7	−98.9	−240.5
	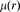	247.4	−81.9	−108.5	−129.7	−431.3	−155.9	328.9	−61.6	−137.4
	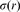	106.3	−112.7	−161.7	−210.2	−2494.6	−278.7	138.1	−82.9	−227.2
HB	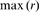	92.0	−131.4	−182.2	−225.5	−791.3	−246.2	112.8	−93.8	−207.3
	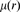	272.8	−82.6	−109.2	−131.3	−432.7	−142.6	366.7	−60.5	−121.9
	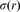	127.8	−112.0	−161.0	−211.0	−2482.7	−238.3	165.5	−79.9	−189.0
ATS	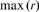	120.7	−137.9	−195.9	−242.1	−854.7	−253.9	143.7	−97.7	−219.7
	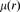	369.8	−87.6	−118.6	−142.1	−469.4	−148.1	488.6	−63.6	−130.6
	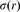	173.0	−118.1	−173.1	−226.1	−2620.6	−241.3	218.5	−83.9	−199.6
DC	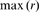	119.2	−143.6	−201.8	−250.1	−882.0	−294.2	143.9	−102.1	−246.5
	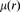	404.6	−89.5	−120.6	−145.5	−482.0	−168.9	540.4	−64.0	−143.9
	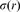	175.7	−121.8	−177.0	−232.0	−2678.1	−288.0	224.7	−86.6	−226.7
OS	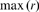	72.9	−258.2	−341.1	−419.4	−1446.2	−539.9	100.0	−205.0	−443.3
	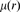	349.3	−148.4	−189.5	−228.6	−754.5	−289.1	486.6	−119.9	−240.5
	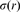	117.7	−203.8	−279.5	−362.9	−3939.7	−514.2	164.6	−160.9	−390.7
MB	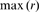	222.1	−221.6	−311.1	−392.2	−1418.5	−470.9	266.4	−155.8	−382.7
	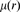	849.5	−137.8	−184.8	−226.4	−765.8	−266.8	1152.2	−98.0	−221.3
	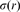	352.8	−184.8	−265.5	−350.9	−3908.0	−444.5	452.7	−130.7	−339.9
U	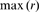	404.3	−200.7	−303.2	−398.6	−1491.0	−481.1	466.6	−120.8	−388.7
	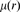	1672.5	−133.7	−190.8	−241.7	−828.3	−285.2	2206.4	−78.8	−235.9
	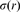	693.6	−175.0	−266.7	−364.3	−4068.4	−462.1	862.0	−107.3	−354.5

Summary of 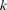, the distance to the mean (in standard deviations), between real values and those of random texts for three different rank statistics: 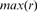 (the maximum rank), 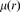 (the mean rank) and 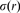 (the standard deviation of the rank). The first column contains the abbreviation of the text (see Table 1 for the meaning of each abbreviation). Texts are sorted by increasing length. The columns after the first column correspond to different versions of the random text model and different parameter settings. For each text and parameter setting, we show 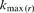, 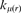 and 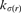, the distances from each of the three rank statistics. 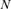 is the number of characters other than space. 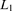 and 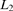 are two parameter settings borrowed from [2]. 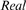 indicates that all character probabilities are obtained from the original text. Distances are computed from the estimated mean and standard deviation of the rank of a certain random text through 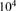 independently generated replicas. The random texts have the same length in words as the target real text.

How can we determine the significance of these distances? The Chebyshev inequality provides us with an upper bound of the, p-value, the probability that the value of the distance is due to mere chance for any kind of distribution. This upper bound is 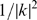, where 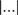 is the absolute value operator [29]. Henceforth we use the term absolute distance to refer to 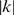. We estimate the mean (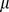) and standard deviation (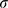) that are needed to compute the distances (Eqs. 6, 7 and 8) by simulating the version of the random text with the parameter setting under consideration a certain number of times (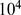 in our case). Table 3 shows, that all absolute distances (for any novel, any version of the random text and any parameter setting) are above 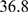. The minimum absolute distance is achieved by 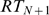 for 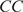 with the parameters setting 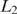 and the statistic 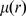. This means that the distance p-values, in all cases do not exceed 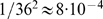. Next we examine some concrete examples of the huge distance between a real text and a certain random text model and parameter setting using the results in Table 3. The minimum absolute distance achieved by any statistic for:

- the fair die rolling experiment considered in [7] (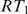 with 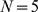) is 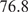 standard deviations, which is achieved by the text 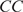 (*A Christmas carol*, by Dickens). This means that the p-value of the differences for any statistic and for all texts does not exceed 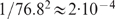. In our version of the model, we do not allow for empty words to make the model more realistic.
- the variant of the random text model considered in [4] (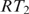) is 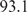 standard deviations, which is achieved by the text AAW (*Alice's adventures in wonderland*, by Carrol). This means that the p-value of the differences for any statistic and for all the texts does not exceed 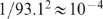. In our version of the model, we do not allow for empty words to make the model realistic and estimate the parameters from the real text.
- the random text with unequal letter probabilities (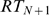 with the three different parameters settings) is 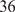 standard deviations, which is achieved by 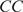 with the parameter setting 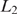 (this is the minimum distance for all versions of the random texts and parameter settings). Thus, the p-value of the differences for all statistics and for all the tests does not exceed 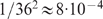. This is striking, since it has been claimed that unequal letter probabilities improve the fit of random texts to the rank distribution of real texts dramatically [25]. In contrast, we show that the hypothesis of a random text is still rejected with unequal letter probabilities.

Next we focus on the sign of the distances in order to shed light on the nature of the disagreement between real and random texts. The sign of the distance indicates whether the actual value is too small (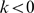) or too large (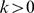) for the hypothesis of a random text. In all the cases shown in Table 3, the sign of this new distance is negative (the real values of the statistic are too small) except for 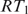 with 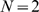 and 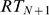 with the parameters setting 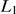, where that distance is positive (the real values of the statistic are too large in these cases). A further statistical test confirming the results obtained thus far is presented in Text S1.

## Discussion

We have seen that three different rank statistics are able to show, independently, that ten English texts and random texts with different versions and parameters settings are statistically inconsistent in all cases. We have seen that for the majority of the parameter settings considered, the nature of the disagreement is that the real rank statistic is smaller than that expected for a random text.

Although we have shown the poor fits of random texts by means of rigorous statistical tests, our limited exploration of the parameter space cannot exclude the possibility that random texts provide good fits for actual rank histograms with parameter values not considered here. Notice that random texts fail both with arbitrarily chosen parameters, e.g., the fair die rolling experiment [7] with 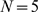 and 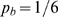 (model 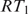), and with parameters inferred from the target text, which would seem *a priori* more likely to yield a good fit. Despite our limited exploration of the parameter space, in the absence of concrete parameter values for which random texts fit real rank histograms accurately, the meaningfulness for natural languages of Zipf's law-like word distributions remains viable.

We believe that the quest for parameters that provide a good fit of random texts on real texts is a tough challenge for detractors of the meaningfulness of Zipf's law, because real writers do not produce words by concatenating independent events under a certain termination probability. Real writers extract words from a mental lexicon that provides almost ‘ready to use’ words [30]. Our main point here is that generally the lexicon provides root word forms that can be completed with affixes. The valid root forms are basically determined *a priori*. Although writing can be a very creative exercise, real writers do not construct words ‘on the fly’ as in the random texts that have previously been presented as an argument against the utility of probability distributions in language. Although some writers do invent many words, their creativity is limited by the need to be understood by their readers. Indeed, the meaning of invented words has to be guessed from the surrounding words. If the context words are also invented, then the reader is likely to get completely lost. Considered from this perspective, random texts are a case of maximum word creativity, and are not limited by a need to be understood (recall the meaningless examples of random texts in the Introduction and the Results section) but only constrained by the prior character probabilities.

There are still many models of Zipf's law for which the goodness of fit to real texts has not been studied rigorously (e.g., [31], [32]). A remarkable exception is [21]. Further research is necessary in order to establish which models provide the best fit in a statistically rigorous sense. Indeed, this is yet another reason to conclude that two fundamental research problems about Zipf's law in natural languages, namely its meaningfulness and a realistic explanation of it, remain open.

## Materials and Methods

### Materials

To simplify the analysis, we normalize the English texts in Table 1 by removing all marks, lower casing all letters, converting all spaces into blanks and leaving only one blank after each word. In this way, we obtain a sequence of words whose length is at least one character and separated by a single blank. A similar normalization procedure is used in [19] although this study does not provide enough details to determine if its normalization procedure is exactly the same as ours.

After text normalization, there is a small fraction of word characters that are not letters in the English alphabet. Most of these characters are digits or accents. To make sure that our results are not a consequence of these infrequent characters we repeated the fitting tests excluding words not made exclusively of English lowercase letters from ‘a’ to ‘z’ after text normalization. We found that the results were qualitatively identical: each of the three rank statistics is able to reject the hypothesis of a random text in all cases.

### Computational Methods

Here we aim to provide some guidelines to perform the computer calculations presented in this article for easy replication of our results. In what follows we consider the computational efficiency of three issues: (i) the generation of random words; (ii) counting the frequency of random words; (iii) and sorting.

#### Random word generation

Here we explain how to generate a random word efficiently. We start with the simplest (or naïve) algorithm of random word generation (we assume that the space delimiting words does not belong to the word):

1. Start with an empty string of characters 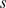.
2. Generate a random character 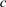 and add it to 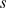.
3. Generate a uniform random deviate 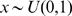.
4. While 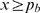 do
5. Generate a random character 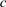 and add it to 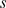.
6. Generate a uniform random deviate 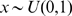.

Generating a uniformly distributed random letter (steps 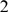 and 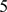) for the models 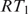 and 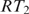 with a standard random uniform given the alphabet size 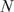 is straightforward. Generating a random letter for 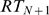 where probabilities come from a real text can also be easily performed in 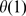 time using a table look-up method [33]. If character probabilities come from a real text (as in the parameter setting 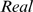 (Results section), we can place all the characters other than space from that text in a table and then choose one uniformly in 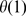 time. This needs space 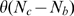, where 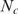 is the number of characters of the text after text normalization (including blanks) and 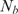 is the number of blanks. If the character probabilities are given *a priori* but are rational numbers (as in the parameter settings 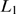 and 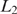 borrowed from [2]), then we can generate a table where the relative frequency of each character is the same as the desired probability. Alternatively, for the case of 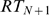 in general, one can use an inversion method with a guided table [33]. The fact that normally 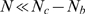 in large enough texts implies that this inversion method requires less space than the table look-up method while keeping the 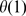 time for generating a random letter.

Imagine that a random word has length 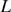 (we assume 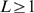 in our random texts). The naïve algorithm above needs invoking a random uniform deviate generator 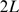 times, i.e. 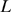 times for generating each of the 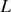 random characters (steps 2 and 5) and 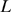 times for determining if more characters have to be added or not (steps 3 and 6). We can reduce the number of random uniform deviates that need to be generated using the following algorithm:

1. Generate a random geometric deviate 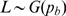.
2. Generate a random word 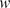 of length 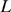,

where is Step 2 is performed through the following algorithm

1. Start with an empty string of characters 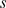.
2. Repeat 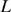 times
3. Generate a random character 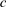 and add it to 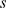.

Of key importance is the generation of the random geometric deviate in 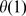 time. It is possible to generate a random geometric deviate 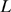 with parameter 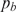 (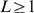) from a random uniform deviate 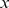 through the formula [33], [34]

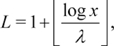
(9)where 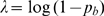. To save computation time, the constant 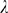 is calculated only once. The second version of the algorithm needs to generate only 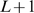 uniform deviates (one for generating the geometric deviate and 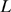 for each of the 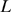 characters comprising the word) whereas the naïve first version required 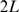 uniform deviates.

It is still possible to generate a random word of length 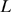 with only 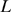 uniform deviates. The idea is to allow the blank to be among the characters that can be generated once the first non-blank character has been placed. The algorithm is

1. Start with an empty string of characters 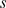.
2. Generate a random character 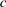 (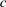 cannot be a blank) and add it to 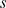.
3. Generate a random character 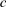 (possibly a blank).
4. While 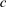 is different than blank do
5. Add 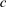 to 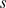.
6. Generate a random character 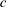 (possibly a blank).

#### Word frequency counting

We define 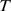 as the length of a text in words. By ignoring the length of a word, the frequency of a word efficiently can be counted in 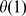 time and 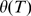 space using a hashing table [35] of character strings.

With simultaneous random word generation and counting, the time efficiency can be improved by employing more memory for the case of 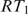 and 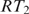. The idea is to keep the hashing table only for counting the frequency of the words of lengths greater than 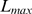 and using a matrix 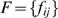 for counting the frequency of each of the 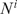 words of length 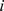 such that 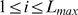. 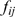 is the frequency of the 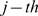 word of length 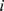 with 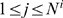. In this way, a random word of length 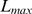 or smaller can be simultaneously generated and counted involving only two random deviates with the following simple algorithm:

1. Generate a random geometric deviate 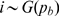

2. If 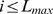 then
3. Generate a random uniform number 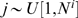.
4. Increase 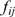 by one.
5. else
6. Generate a random word 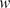 of length 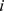 by means of the algorithm above.
7. Increase the frequency of 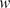 by updating the hashing table of character strings.

The extra memory needed for the table of words of length not exceeding 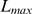 is
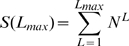
(10)

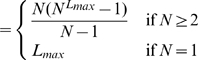
(11)


#### Sorting

Sorting natural numbers efficiently is needed to calculate ranks. Obtaining the ranks of a certain text (real or random) requires sorting the word frequencies from the random text in decreasing order. All the above techniques may not contribute to increase significantly the speed of the computer calculations if the sorting takes more than 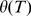 time, where 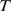 is the length in words of the text. In our case, we can take advantage of the fact that frequencies lie within the interval 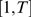 and then we can use counting sort [35], which allows one to sort elements in linear time.

## Supporting Information

Text S1 (0.24 MB PDF)
