# Supplementary material for: Random Texts Do Not Exhibit the Real Zipf's Law-Like Rank Distribution
Source: PLoS One. 2010 Mar 9;5(3):e9411. doi: 10.1371/journal.pone.0009411 (PMC2834740; doi:10.1371/journal.pone.0009411)
Supplement: Text S1 — (0.24 MB PDF) [file pone.0009411.s001.pdf]

Supporting Information

Ramon Ferrer-i-Cancho[1,*], Brita Elvevåg[2]
1 Departament de Llenguatges i Sistemes Informàtics, Universitat Politècnica de Catalunya, Barcelona, Catalonia, Spain,
2 Clinical Brain Disorders Branch, National Institute of Mental Health, National Institutes of Health, Bethesda, Maryland, USA
∗ Corresponding author - Email: rferrericancho@lsi.upc.edu

1 The minimum word length in Li's article

Li [1] does not explicitly assume that the minimum word length that his random text model generates is one. The fact that he shows examples of random texts (p. 1842 of [1]) with sequences of more than one blank in a row at the beginning of his article can be confusing because there is evidence later in the same article that the author is implicitly assuming that words have a minimum length of one. In Eqs. 3 and 15 of [1], summations are restricted to lengths greater or equal than one. Further evidence of the absence of empty words in the simulations comes from Fig. 1 of [1]. There, the plots of the rank spectrum for different alphabet sizes of a random text with equal character probabilities start with plateaus of the number of characters in the alphabet (excluding the space), confirming the absence of empty words. However, the manner in which the parameters of the simulations with unequal character probabilities are presented is confusing. We assume that we have N characters other than space and $p_1, ..., p_i, ..., p_N$ are the probabilities of each these characters in Li's model and p_b is the probability of blank. The presentation by Li of these probabilities allows one to interpret - although incorrectly from what we have noted above - that the probability of a blank does not depend upon the number of characters that have already been placed for the current word. In contrast, if the current word does not have any characters the probability of a blank is actually zero and the probabilities for other characters are no longer valid. For this reason, it would be more accurate to state that $p_b, p_1, ..., p_N$ are the character probabilities when the word being constructed has more than one character. If not, then

- The probability of a non-blank character labeled with i (for $1 \leq i \leq N$) is

$$p'_i = \frac{p_i}{\sum_{i=1}^{N} p_i} = \frac{p_i}{1 - p_b}. \quad (1)$$

- The probability of a blank is zero.

2 Supplementary figures

In our main article, the plots of the rank histograms showed 3σ upper and lower bounds for random texts. When ranks are large enough, the lower bounds cannot be shown because the lower bound is negative and plots are on a logarithmic scale. To overcome the limits of these plots for sufficiently large ranks, Figs. 1, 2 and 3 show an estimate of the expected frequency of each rank.

3 Supplementary tables

To gain a general overview of the significance of the distance to the mean k for any statistic, any version of the random text and any parameter setting considered in this article, it is useful to consider, k^* the critical value of $|k|$ for which the Chebyshev inequality warrants that k is statistically significant at a certain significance level x. It is easy to see that $k^* = x^{-1/2}$. Some critical values are shown in Table 1.

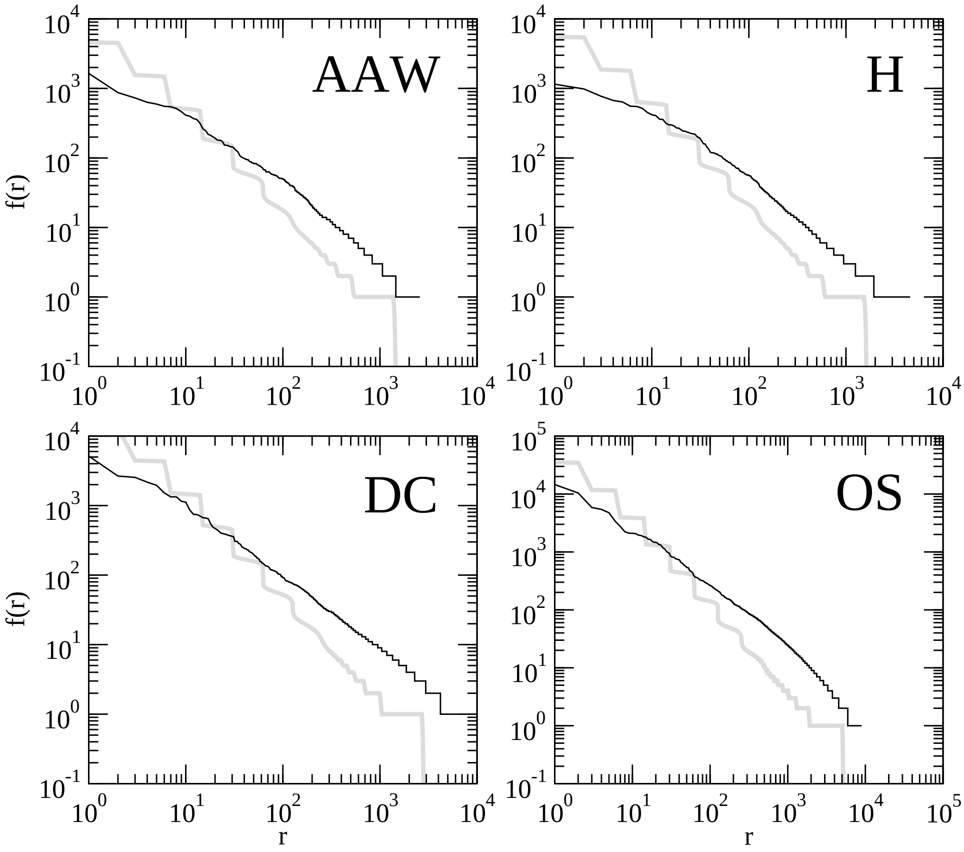

Figure 1. The rank histograms of English texts versus that of random texts (RT_1). A comparison of the real rank histogram (thin black line) and the expected histogram of a random text of the same length in words (thick gray line) involving four English texts. $f(r)$ is the frequency of the word of rank r. For the random text we use the model RT_1 with alphabet size $N = 2$. The expected histogram of the random text is estimated averaging over the rank histograms of 10^4 random texts. For ease of presentation, the expected histogram is cut off at expected frequencies below 0.1. AAW: *Alice's adventures in Wonderland*. H: *Hamlet*. DC: *David Crockett*. OS: *The origin of species*.

Table 1. Critical values of the absolute distance.

x	k^*
0.05	$2\sqrt(5) \approx 4.47$
0.01	10
0.001	$10\sqrt(10) \approx 31.62$
0.0001	100

Critical absolute distance, k^*, versus significance level, x, for some representative significance levels.

The distances to the mean in standard deviations are computed for three rank statistics, i.e. $\max(r)$

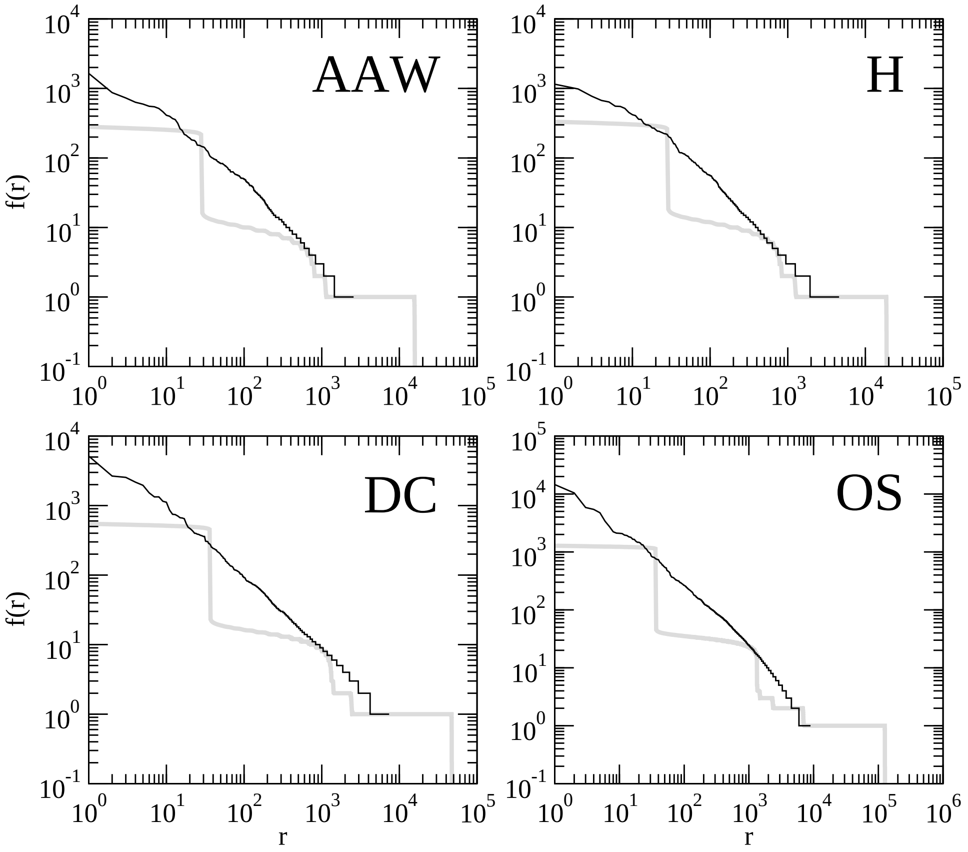

Figure 2. The rank histograms of English texts versus that of random texts (RT_2). The same as Fig. 1 for the model RT_2 with alphabet size N and probability of blank p_b obtained from the real text.

(the maximum rank rank), $\mu(r)$ (the mean rank) and $\sigma(r)$ (the standard deviation of the rank). These distances are obtained from the mean and the standard deviation of these statistics, which are estimated numerically. Table 2 shows the estimated mean and the standard deviation of $\max(r)$. Tables 3 and 4 show, respectively, the same for $\mu(r)$ and $\sigma(r)$.

4 A further statistical test

The distance tests that we have employed in the main article are eminently suitable in terms of showing the separation between real texts and random texts. However, they have two clear drawbacks: (a) the distances are computed from estimates of the mean and the variance while the Chebyshev inequality requires that true mean and variance are used in order to be exact (b) Chebyshev inequality provides only an upper bound of the p-value so that if the method is taken as the ultimate answer about the goodness of fit a random text, type II errors (i.e. failures to distinguish a random text from a real texts when there is actually a significant difference) can be made. Notice that problem (b) is not especially of concern for our current results because we have already been able to reject the hypothesis of a random

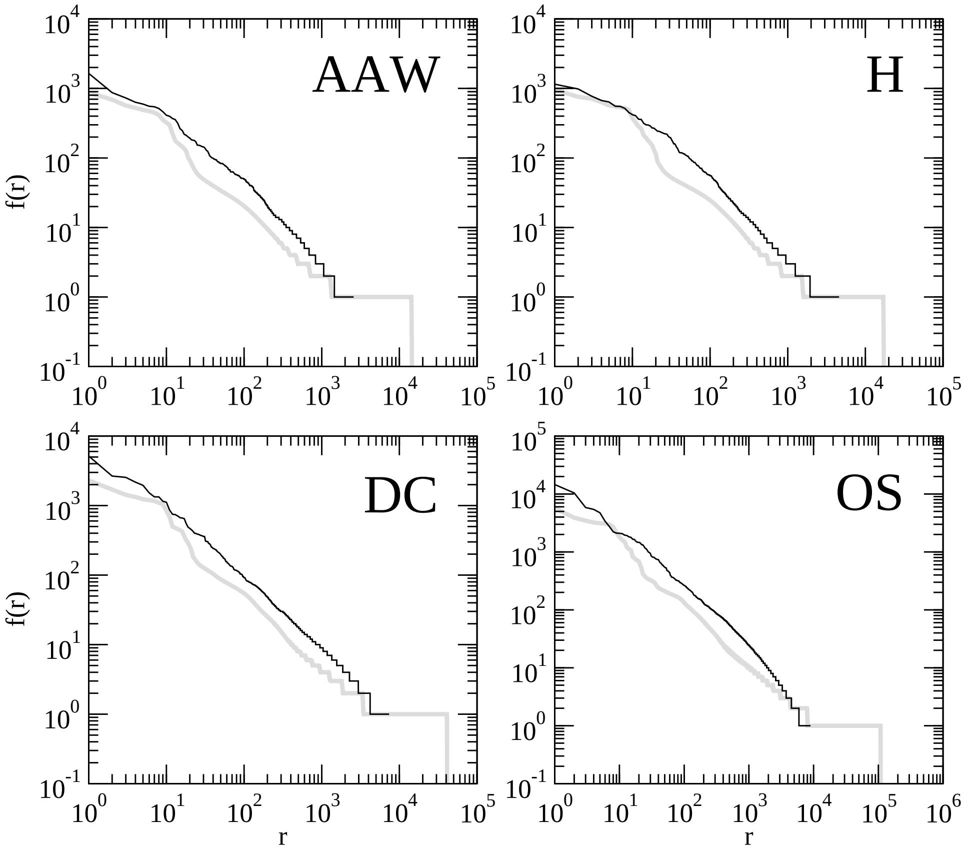

Figure 3. The rank histograms of English texts versus that of random texts (RT_{N+1}). The same as Fig. 1 for the model RT_{N+1} with alphabet size N and character probabilities obtained from the real text.

text with our approximate method based on distances to the mean, but this issue may be crucial in future studies in which differences between real and random texts are not so radical.

A way of bypassing these drawbacks is to estimate the actual p-values rather than an approximate upper bound. Ultimately, our goal is to establish if the values of a certain statistic x ($max(r)$, $\mu(r)$ or $\sigma(r)$) are significantly different from the values obtained from a random text. We use x_{RT} to refer to the value of the statistic x obtained in a random text of the same length as the target real text. In this way, we can define the probability that x_{RT} is equal or greater than a real value x, i.e. $p(x_{RT} \geq x)$ and the probability that x_{RT} is equal or smaller than a real value x, i.e. $p(x_{RT} \leq x)$. $p(x_{RT} \leq x)$ is the left p-value and $p(x_{RT} \geq x)$ is the right p-value [2]. If one of these two probabilities is equal or smaller than the significance level, then there is a statistically significant difference between x and the values of x_{RT} produced by a random text. We estimate the probability of the left and right p-values by generating 10^4 random texts of the same length as the target real text.

With this more accurate method, we can confirm the results in our main article. In particular, we find that there is no random text with the parameters setting considered in our article that is statistically consistent with a real text for the three different rank statistics considered in our article (Tables 5, 6 and 7). In all cases, we find only two situations: (a) the estimated left p-value is 0 and the estimated

Table 2. The maximum rank in random texts.

		RT_1					RT_2	RT_{N+1}		
Abbrv.		$N = 2$	$N = 4$	$N = 5$	$N = 6$	$N = 26$	-	L_1	L_2	*Real*
AAW	μ	1412.37	9794.42	13217.10	15817.13	25870.57	15706.88	1211.55	7514.15	14370.40
	σ	27.26	74.03	79.89	81.04	40.63	81.76	25.23	66.49	80.22
CC	μ	1474.11	10381.41	14045.01	16831.02	27654.18	17505.16	1263.46	7950.47	15936.98
	σ	28.14	75.78	81.52	83.18	42.12	83.54	25.68	68.69	83.82
H	μ	1585.63	11467.56	15583.34	18718.72	30996.93	18671.36	1356.42	8754.14	17114.37
	σ	28.95	79.54	86.22	89.26	45.43	89.37	26.55	71.17	88.07
ECHU	μ	2269.99	18704.61	25972.60	31570.14	54301.45	36742.73	1925.14	14053.56	32697.43
	σ	34.96	103.05	114.07	117.94	62.08	115.38	31.87	92.41	115.52
HB	μ	2319.56	19261.41	26780.28	32577.46	56159.26	35250.43	1965.65	14457.90	30338.68
	σ	35.31	104.17	116.40	119.79	63.93	120.54	31.94	94.81	119.52
ATS	μ	2637.79	22961.07	32159.36	39307.77	68684.86	40957.10	2228.51	17134.03	36154.56
	σ	37.54	114.54	127.55	132.74	71.97	133.08	34.37	101.95	131.91
DC	μ	2756.62	24381.16	34229.23	41912.30	73573.14	47137.94	2326.48	18156.71	41076.01
	σ	38.82	118.36	133.05	138.05	75.04	135.13	35.15	105.45	136.66
OS	μ	5105.23	56502.66	82230.26	102993.58	193327.53	126113.68	4242.27	40941.22	108385.52
	σ	52.84	184.17	214.82	224.21	127.49	217.01	47.15	156.02	224.28
MB	μ	5248.14	58668.63	85527.27	107216.27	201879.54	123150.65	4358.44	42464.29	104401.99
	σ	53.77	187.22	219.69	229.52	130.20	225.01	48.17	162.20	227.91
U	μ	5992.11	70301.53	103307.15	130052.94	248538.46	150654.60	4959.98	50581.82	128299.54
	σ	57.43	204.75	244.38	253.00	147.10	252.43	51.98	176.93	254.90

Summary of the statistics of $\max(r)$, the maximum rank in a random text. The first column contains the abbreviation of the text. Texts are sorted by increasing length. The columns after the first column correspond to different versions of the random text model and different parameter settings. Every two rows correspond to a different text. For each text and parameter setting, we show $\mu(\max(r))$ (top) and $\sigma(\max(r))$ (bottom), which are, respectively, the mean and the standard deviation of $\max(r)$. N is the number of characters other than space. L_1 and L_2 are two parameter settings borrowed from [1]. *Real* indicates that all character probabilities are obtained from the original text. $\mu(\max(r))$ and $\sigma(\max(r))$ are estimated through 10^4 independently generated replicas. The random texts have the same length in words as the target real text.

right p-value is 1 (ie. the actual value is significantly small) or (a) the estimated left p-value is 1 and the estimated right p-value is 0 (i.e. the actual value is significantly large). Thus, we can accurately distinguish, by means of a single rank single statistic, a real text in our dataset from a random text with any of the parameter settings considered in this article.

References

1. Li W (1992) Random texts exhibit Zipf's-law-like word frequency distribution. IEEE T Inform Theory 38: 1842-1845.

2. Conover WJ (1999) Practical nonparametric statistics. New York: Wiley. 3rd edition.

Table 3. The mean rank in random texts.

		RT_1					RT_2	RT_{N+1}		
Abbrv.		$N=2$	$N=4$	$N=5$	$N=6$	$N=26$	-	L_1	L_2	*Real*
AAW	μ	58.24	1825.74	3260.23	4629.58	12242.65	4578.32	45.50	1096.92	3836.38
	σ	1.50	26.32	38.25	46.44	38.31	46.44	1.21	18.20	41.78
CC	μ	59.37	1917.23	3440.83	4899.84	13075.14	5306.46	46.30	1147.76	4401.98
	σ	1.51	26.67	38.76	47.42	39.68	49.41	1.20	18.58	45.24
H	μ	61.32	2084.09	3773.10	5399.05	14633.37	5381.09	47.66	1239.66	4528.93
	σ	1.49	27.56	40.51	50.43	42.73	50.33	1.18	18.91	45.50
ECHU	μ	71.87	3141.76	5937.72	8701.80	25445.30	11744.22	55.01	1810.46	9320.48
	σ	1.46	33.05	50.71	63.76	57.98	72.56	1.15	22.36	64.69
HB	μ	72.56	3219.97	6101.34	8955.42	26304.38	10481.36	55.48	1851.92	7787.36
	σ	1.47	33.26	51.56	64.60	59.70	70.19	1.14	22.79	59.98
ATS	μ	76.77	3731.73	7176.87	10633.00	32091.66	11524.14	58.38	2121.48	9024.60
	σ	1.45	35.60	55.33	70.50	67.06	73.68	1.13	23.72	64.41
DC	μ	78.27	3924.77	7584.43	11276.09	34347.81	14216.75	59.41	2222.23	10838.72
	σ	1.46	36.39	57.34	72.92	69.87	80.23	1.13	24.27	70.67
OS	μ	102.37	7944.99	16493.58	25659.01	89358.57	38237.81	75.59	4258.18	28367.64
	σ	1.40	49.58	83.91	109.68	117.65	130.24	1.06	30.60	115.50
MB	μ	103.60	8199.49	17079.23	26617.50	93271.59	34958.10	76.42	4384.81	25257.01
	σ	1.40	50.12	85.42	111.88	120.11	126.18	1.05	31.57	108.28
U	μ	109.71	9542.93	20196.51	31747.89	114589.00	42418.60	80.45	5043.15	30904.48
	σ	1.38	53.24	93.12	121.31	135.42	140.24	1.06	33.20	120.73

Summary of the statistics of $\mu(r)$, the mean rank in a random text. For every text and parameter setting, we show $\mu(\mu(r))$ (top) and $\sigma(\mu(r))$ (bottom), which are, respectively, the mean and the standard deviation of $\mu(r)$. The format of the table is the same as that of Table 2. $\mu(\mu(r))$ and $\sigma(\mu(r))$ are estimated through 10^4 independently generated replicas.

Table 4. The standard deviation of rank in random texts.

		RT_1					RT_2	RT_{N+1}		
Abbrv.		$N=2$	$N=4$	$N=5$	$N=6$	$N=26$	-	L_1	L_2	*Real*
AAW	μ	185.39	2857.51	4190.83	5181.42	7824.44	5130.35	148.82	2001.03	4636.93
	σ	5.00	28.65	31.15	30.07	3.62	30.46	4.29	24.36	30.77
CC	μ	191.32	3020.29	4447.36	5510.32	8369.14	5745.51	153.38	2108.96	5175.86
	σ	5.10	29.31	31.83	30.93	3.80	30.51	4.32	25.11	31.84
H	μ	201.81	3320.03	4922.78	6121.87	9390.82	6096.19	161.31	2306.24	5515.94
	σ	5.14	30.69	33.72	33.32	4.19	33.40	4.36	25.88	33.86
ECHU	μ	262.29	5282.64	8102.70	10265.13	16536.09	12143.29	206.89	3576.53	10691.23
	σ	5.64	39.24	44.78	44.75	6.28	40.48	4.74	32.75	43.27
HB	μ	266.46	5431.53	8347.94	10588.72	17106.93	11565.13	209.95	3671.60	9732.85
	σ	5.66	39.64	45.70	45.48	6.49	44.38	4.72	33.55	46.26
ATS	μ	292.56	6415.46	9975.09	12746.79	20957.67	13371.96	229.43	4295.94	11533.88
	σ	5.81	43.34	50.13	50.63	7.50	50.04	4.89	35.72	51.28
DC	μ	302.10	6790.71	10598.47	13580.38	22461.51	15523.54	236.53	4532.39	13260.63
	σ	5.94	44.72	52.29	52.74	7.88	49.23	4.94	36.81	52.55
OS	μ	471.82	15013.18	24804.21	32920.83	59411.66	41651.95	360.80	9588.56	35005.13
	σ	6.82	67.42	84.19	87.20	14.76	78.53	5.55	51.68	86.32
MB	μ	481.30	15554.42	25767.17	34246.33	62056.61	40346.15	367.72	9916.61	33153.94
	σ	6.88	68.42	86.09	89.32	15.13	84.22	5.62	53.61	88.97
U	μ	529.51	18442.07	30938.53	41397.08	76499.65	49295.80	402.54	11645.82	40718.16
	σ	7.09	74.28	95.58	98.68	17.46	94.89	5.85	57.77	99.51

Summary of the statistics of $\sigma(r)$, the standard deviation of rank in a random text. For every text and parameter setting, we show $\mu(\sigma(r))$ (top) and $\sigma(\sigma(r))$ (bottom), which are, respectively, the mean and the standard deviation of $\sigma(r)$. The format of the table is the same as that of Table 2. $\mu(\sigma(r))$ and $\sigma(\sigma(r))$ are estimated through 10^4 independently generated replicas.

Table 5. Estimated left and right p-values for the vocabulary size.

		RT_1					RT_2	RT_{N+1}		
Abbrv.	p-value	$N = 2$	$N = 4$	$N = 5$	$N = 6$	$N = 26$	-	L_1	L_2	*Real*
AAW	*left*	1	0	0	0	0	0	1	0	0
	right	0	1	1	1	1	1	0	1	1
CC	*left*	1	0	0	0	0	0	1	0	0
	right	0	1	1	1	1	1	0	1	1
H	*left*	1	0	0	0	0	0	1	0	0
	right	0	1	1	1	1	1	0	1	1
ECHU	*left*	1	0	0	0	0	0	1	0	0
	right	0	1	1	1	1	1	0	1	1
HB	*left*	1	0	0	0	0	0	1	0	0
	right	0	1	1	1	1	1	0	1	1
ATS	*left*	1	0	0	0	0	0	1	0	0
	right	0	1	1	1	1	1	0	1	1
DC	*left*	1	0	0	0	0	0	1	0	0
	right	0	1	1	1	1	1	0	1	1
OS	*left*	1	0	0	0	0	0	1	0	0
	right	0	1	1	1	1	1	0	1	1
MB	*left*	1	0	0	0	0	0	1	0	0
	right	0	1	1	1	1	1	0	1	1
U	*left*	1	0	0	0	0	0	1	0	0
	right	0	1	1	1	1	1	0	1	1

Summary of left and right p-values for the rank statistic $max(r)$ (the maximum rank). For each real text and parameter setting, we show the left p-value (top) and right p-value (bottom). p-values are estimated over 10^4 independently generated random texts, which have the same length in words as the target real text. For further details refer to Table 2.

Table 6. Estimated left and right p-values for the mean rank.

		RT_1					RT_2	RT_{N+1}		
Abbrv.	p-value	$N = 2$	$N = 4$	$N = 5$	$N = 6$	$N = 26$	-	L_1	L_2	*Real*
AAW	*left*	1	0	0	0	0	0	1	0	0
	right	0	1	1	1	1	1	0	1	1
CC	*left*	1	0	0	0	0	0	1	0	0
	right	0	1	1	1	1	1	0	1	1
H	*left*	1	0	0	0	0	0	1	0	0
	right	0	1	1	1	1	1	0	1	1
ECHU	*left*	1	0	0	0	0	0	1	0	0
	right	0	1	1	1	1	1	0	1	1
HB	*left*	1	0	0	0	0	0	1	0	0
	right	0	1	1	1	1	1	0	1	1
ATS	*left*	1	0	0	0	0	0	1	0	0
	right	0	1	1	1	1	1	0	1	1
DC	*left*	1	0	0	0	0	0	1	0	0
	right	0	1	1	1	1	1	0	1	1
OS	*left*	1	0	0	0	0	0	1	0	0
	right	0	1	1	1	1	1	0	1	1
MB	*left*	1	0	0	0	0	0	1	0	0
	right	0	1	1	1	1	1	0	1	1
U	*left*	1	0	0	0	0	0	1	0	0
	right	0	1	1	1	1	1	0	1	1

Summary of left and right p-values for the rank statistics $\mu(r)$ (the mean rank). For each real text and parameter setting, we show the left p-value (top) and right p-value (bottom). p-values are estimated over 10^4 independently generated random texts, which have the same length in words as the target real text. For further details refer to Table 2.

Table 7. Estimated left and right p-values for the standard deviation of the ranks.

		RT_1					RT_2	RT_{N+1}		
Abbrv.	p-value	$N = 2$	$N = 4$	$N = 5$	$N = 6$	$N = 26$	-	L_1	L_2	*Real*
AAW	*left*	1	0	0	0	0	0	1	0	0
	right	0	1	1	1	1	1	0	1	1
CC	*left*	1	0	0	0	0	0	1	0	0
	right	0	1	1	1	1	1	0	1	1
H	*left*	1	0	0	0	0	0	1	0	0
	right	0	1	1	1	1	1	0	1	1
ECHU	*left*	1	0	0	0	0	0	1	0	0
	right	0	1	1	1	1	1	0	1	1
HB	*left*	1	0	0	0	0	0	1	0	0
	right	0	1	1	1	1	1	0	1	1
ATS	*left*	1	0	0	0	0	0	1	0	0
	right	0	1	1	1	1	1	0	1	1
DC	*left*	1	0	0	0	0	0	1	0	0
	right	0	1	1	1	1	1	0	1	1
OS	*left*	1	0	0	0	0	0	1	0	0
	right	0	1	1	1	1	1	0	1	1
MB	*left*	1	0	0	0	0	0	1	0	0
	right	0	1	1	1	1	1	0	1	1
U	*left*	1	0	0	0	0	0	1	0	0
	right	0	1	1	1	1	1	0	1	1

Summary of left and right p-values for the rank statistic $\sigma(r)$ (the standard deviation of the rank). For each real text and parameter setting, we show the left p-value (top) and right p-value (bottom). p-values are estimated over 10^4 independently generated random texts, which have the same length in words as the target real text. For further details refer to Table 2.
